# Comparison of the efficacy of two interventions in ameliorating abdominal thickness and sitting function in children with diplegia

**DOI:** 10.1016/j.jtumed.2022.01.011

**Published:** 2022-02-12

**Authors:** Mostafa S. Ali, Ahmed S. Awad

**Affiliations:** Physical therapy for pediatrics, Faculty of Physical Therapy, Cairo University, Egypt

**Keywords:** العلاج بمساعدة الخيول, العلاج بركوب الخيل, الشلل الدماغي, اهتزاز الجسم بالكامل, سماكة عضلات البطن, وظيفة الجلوس, شلل مزدوج, التصوير بالموجات فوق الصوتية, Abdominal thickness, Diplegia, Hippotherapy, Sitting function, Whole-body vibration

## Abstract

**Objective/Background:**

The abdominal muscles are extremely important in stabilizing the trunk and maximizing postural stability. The presence of apparent stiffness in children with spastic diplegia is associated with unsteadiness, impaired walking, and pelvic malrotation. The aim of this study was to compare the efficacy of hippotherapy and whole-body vibration in ameliorating abdominal muscle thickness and sitting function in children with diplegia.

**Methods:**

A total of 60 children with spastic diplegia were selected from the Faculty of Physical Therapy's outpatient clinic, Cairo University, and randomly allocated into two groups. Group A received conventional physical therapy for 1 h in addition to whole-body vibration, whereas group B received hippotherapy for 40 min. The same designed physical therapy program was administered for 12 weeks, three times per week, in both groups. Ultrasonography was used to measure abdominal thickness, and Gross Motor Function Measurement-88 was used to measure functional ability.

**Results:**

A significant improvement in abdominal muscle thickness and sitting function (*p* < 0.05) was observed in both groups, and greater improvements were observed in group B.

**Conclusion:**

Whole-body vibration and hippotherapy training may be recommended to facilitate sitting function and ameliorate abdominal thickness in children with diplegia. Hippotherapy is more effective than whole-body vibration in improving sitting function and abdominal muscle thickness.

## **الملخص**

**أهداف البحث:**

عضلات البطن مهمة للغاية لتحقيق الاستقرار في الجذع وزيادة وضعية الثبات. ارتبط وجود تصلب واضح عند الأطفال المصابين بالشلل النصفي التشنجي بعدم الثبات وضعف المشي وسوء دوران الحوض. كان الهدف من الدراسة هو المقارنة بين فعالية العلاج بركوب الخيل واهتزاز الجسم بالكامل على سماكة عضلات البطن ووظيفة الجلوس عند الأطفال المصابين بشلل مزدوج.

**طرق البحث:**

تم اختيار ستين طفلا مصابا بالشلل النصفي التشنجي من العيادة الخارجية بكلية العلاج الطبيعي بجامعة القاهرة وتم تقسيمهم عشوائيا إلى مجموعتين (أ ، ب). نفذت المجموعة (أ) برنامج العلاج الطبيعي التقليدي لمدة ساعة واحدة بالإضافة إلى اهتزاز الجسم بالكامل بينما أجرت المجموعة (ب) العلاج بركوب الخيول لمدة 40 دقيقة مع نفس برنامج العلاج الطبيعي المصمم لمدة 12 أسبوعا، 3 مرات كل أسبوع لكلا المجموعتين. تم استخدام الموجات فوق الصوتية لقياس سمك البطن وقياس الوظيفة الحركية الإجمالية -88 لقياس القدرة الوظيفية.

**النتائج:**

أظهرت النتائج أن هناك تحسنا ملحوظا في سمك عضلات البطن ووظيفة الجلوس في كلا المجموعتين، مع تحسن أكبر لصالح المجموعة (ب).

**الاستنتاجات:**

قد يقترح تدريب اهتزاز الجسم بالكامل والعلاج بركوب الخيول ليكون تطبيقا جيدا لتسهيل وظيفة الجلوس وتغيير سماكة البطن عند الأطفال المصابين بشلل نصفي. كان العلاج بركوب الخيل أكثر فعالية من اهتزاز الجسم بالكامل في تحسين وظيفة الجلوس وسماكة عضلات البطن.

## Introduction

Cerebral palsy (CP) is a non-progressive lesion or impairment of the developing brain, as defined by abnormalities in autonomic movement and posture.^1^ The degree, category, and locations of lesions in the central nervous system (CNS), as well as the ability of the CNS to adapt or respond through reorganization, determine the clinical signs.^2^ Spastic type diplegia accounts for 44% of all cases and 80% of cases in preterm newborns.^3^ Spastic diplegia affects children and causes substantial trunk paralysis, as well as stiffness in the extremities and motor difficulties.^4^

The abdominal muscles are essential in trunk–pelvic stabilization and postural stability.^5^ The abdominal muscles comprise the internal oblique (IO) and external oblique (EO) muscles, the rectus abdominis (RA), and the transversus abdominis (TA). Postural stability impairment is a prominent symptom of motor disability in children with spastic CP.^6^

Mechanical oscillation, defined by amplitude and frequency, generates a force that acts on the whole body; therefore, low-amplitude whole-body vibration (WBV) may be used for balance and proprioceptive training.^7^^,^^8^ WBV is an approach for increasing muscular strength that can be used in a variety of practical areas.^9^^,^^10^ WBV is performed with the child sitting in a static position on a device. Although each abdominal muscle works individually and has primary stabilizing and motor functions, proficient stability and movement of the trunk require accumulated exertion of the trunk musculature.^11^^,^^12^

Hippotherapy is an integrated exercise program that provides opportunities for balance and strength-building requiring the correct posture to be maintained through the three-dimensional movements of the horse.^13^ In hippotherapy, a physical or occupational therapist controls the horse to influence the child's posture, balance, coordination, strength, and sensorimotor systems, while the child interacts with the horse and responds to its movement.^14^

The visual, vestibular, and somatosensory systems together form a multisensory system that helps children with CP improve their balance and maintain their posture. The CNS, together with the musculoskeletal and cerebellar systems controls these systems, which are influenced by experiences and the environment.^15^ Therefore, therapies such as hippotherapy may diminish the disease's clinical manifestations while also improving postural balance. Previous studies have shown that hippotherapy can improve balance and daily functional activities in children with CP.^16^, ^17^, ^18^, ^19^

When riding a horse, the horse's motions affect the rider, thus causing the rider to undergo a variety of postural changes. Subtle collaborative motions of the rider's neck, torso, and limb muscles are needed to accommodate to the horse's rhythmic movement.^20^ Hippotherapy has been shown to improve muscle symmetry in the trunk and hip, and to decrease asymmetry in the adductor muscles, in the short term. It has also been found to improve vestibular and proprioceptive stimulation, as well as to increase body awareness.^21^ Studies on the effects of hippotherapy in children with CP have recently indicated improvements in gross motor function, postural control, balance, and gait.^22^

The skeletal muscle's force-generating ability, and thus its strength, is a complex function of various features of the muscle architecture, including muscle thickness.^23^ The muscle thickness is the distance between the superficial aponeurosis and the deep aponeurosis in the middle of an ultrasound image at a 90° angle from the deep aponeurosis.^24^ Ultrasonography is a non-invasive technique for determining changes in muscle thickness throughout activation, and it was originally used to assess myocardial activity.^25^

Understanding the efficacy of WBV versus hippotherapy on abdominal muscle thickness and sitting function can help physiotherapists determine which treatment modality WBV or hippotherapy will provide the most benefit in rehabilitation programs designed for children with spastic diplegia. Therefore, the purpose of this study was to compare the effects of WBV and hippotherapy on abdominal muscle thickness and sitting function in children with spastic diplegia, during an intervention period of 12 weeks.

## Materials and Methods

### Study design

This study was a comparative study.

### Subjects

**Figure 1:**
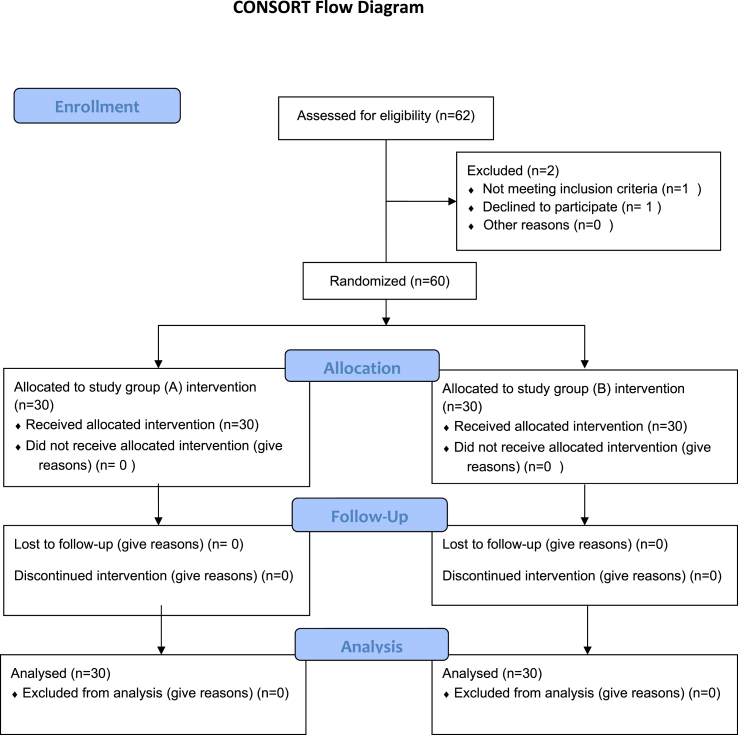
Flowchart of patient randomization.

This study was conducted between February and July 2021. A total of 60 children with spastic diplegia were recruited from the Faculty of Physical Therapy's outpatient clinic at Cairo University. No children from either study group dropped out during treatment, as shown in Figure 1. The inclusion criteria were as follows: (a) children diagnosed with spastic diplegia, (b) average age of 3–5 years, (c) interest in riding, (d) parental consent to participate without changing current therapy, and (e) spasticity ranging from 1 to 1+ according to the Modified Ashworth Scale.^26^ The Gross Motor Function Classification Scale (GMFCS) was used to determine the level of motor function.^27^ Only children in levels III and IV were included. Children who (a) refused to adhere to the study protocol, (b) had deformities of the hip or spine, (c) had experienced surgery of the anterior abdominal wall or took medication during the prior 6 months to decrease spasticity were excluded from the study. In addition, obese children (BMI >25 kg m^−2^) were excluded because considerable adipose tissue impedes ultrasound detection of thickness.^28^

### Randomization

Children were recruited from the clinic and divided equally into two groups. Group A underwent a conventional physical therapy program in addition to WBV, and group B underwent hippotherapy with the similar physical therapy program for 12 weeks. Signed written consent for participation in the study was obtained.

### Procedures

#### Ultrasonography

The thickness of four abdominal muscles was measured with ultrasonography (device type GE Logiq P6) with a frequency of 7.5 MHz. The principal investigator held the transducer head while an assistant gave the child instructions.^29^ The probe was placed 2 or 3 cm from the midline, using the umbilicus as a landmark, then was moved in a semi-circular motion until the deepest image on the screen, the TA, was visible. To confirm this position and measure the thickness, a skin marker pen was used. The probe was then moved in an oblique manner to detect the thickness of the EO, IO, RA, and TA. A large amount of contact gel was used, and the probe pressure was adjusted to obtain optimal values of muscle thickness. After image capture, a vertical line was drawn between the superficial and deep aponeurosis to determine muscle thickness. The investigator collected three measurements along the muscle in different areas, then recorded the average.

#### Gross motor function measurement-88 (GMFM-88)

GMFM-88, a standard measure for children with CP, was used to evaluate the level of motor function in children. The measure involves 88 items in five dimensions: lying and rolling; sitting; crawling and kneeling; standing; and walking, running, and jumping. GMFM-88 is recommended to evaluate the motor level in children. In the current study, GMFM-88 was used to measure the sitting domain (sitting with support) in selected children. The researcher initially positioned each child in a forward sitting position with support on one or both hands, according to the child's abilities, then followed the remaining tasks of the sitting domain. The researcher finally calculated the score by dividing the number of achieved tasks by the total number of sitting domain tasks and multiplying by 100 to obtain the achieved task percentage, which was recorded and saved.^30^

#### Intervention

##### Conventional physical therapy program

A total of 60 children received conventional physical therapy based on neurodevelopmental techniques, which included the following: (a) righting and equilibrium reactions from various positions on a roll and ball to develop postural mechanisms, (b) saving reactions to protect the child from falling over, (c) balance training with a balance board, (d) vestibular training, (e) postural reaction facilitation, (f) facilitation of delayed milestones, particularly sitting, standing, and weight shifting for 1 h, six times per week for 12 weeks).

##### Group A

A total of 30 children underwent the same conventional physical therapy program for 12 weeks in addition to WBV for 10 min.

##### WBV application technique

The children assumed a full squatting position on a vibration platform. The apparatus was set at a frequency of 30 Hz, an amplitude of 2 mm, and a duration of 5 min. The children were instructed to remain in squatting position after the vibration started and to report any discomfort that arose. At the end of 5 min, the vibration turned off automatically. Thereafter, the children rested for 1 min. They were then asked to stand on the vibration platform while supported by the therapist for 5 min, with the same parameters as those used in the squatting position. The total time of application of WBV in each session was 10 min^31^.

##### Group B

A total of 30 children underwent the same conventional physical therapy program for 12 weeks, consisting of 1 h of therapy three times per week, plus 40 min of horseback riding stimulation. The hippotherapy program was performed on a horse. In the initial 5 min warm-up stage, the horse walked around the 45-m-long, 20-m-wide arena with the child facing forward on the horse to become familiar with the horse's width and movement. During the next 5 min, the child faced backward with the horse walking on the left rein, and stopped at the middle of each long side for a 20-s rest. In the final 5 min, the child faced backward with the horse changed to the right rein. The core exercise lasted 20 min and consisted of regular walking, stepping on a stirrup, and fast walking. The final 5 min training was completed by stretching of the lower body.^32^

### Statistical methods

Descriptive statistics and unpaired t-tests were used to compare ages between groups. Chi-squared test was used to compare the sex distribution between groups. Shapiro–Wilk test was used to verify the normal distribution of the data. Levene's test for homogeneity of variances was performed to ensure homogeneity between groups. Mixed design MANOVA was performed to compare effects within and between groups on abdominal muscle thickness and GMFM-88. Post-hoc tests with Bonferroni correction were performed for subsequent multiple comparisons. The level of significance for all statistical tests was set at *p* < 0.05. All statistical analyses were conducted in Statistical Package for Social Studies (SPSS) version 25 for Windows (IBM SPSS, Chicago, IL, USA).

## Results

### Participant characteristics

Table 1 shows the mean ± SD of the ages of participants in the hippotherapy and WBV groups. The mean age did not differ significantly between groups (*p* < 0.05). In addition, no statistically significant difference in the sex distribution was observed between groups (*p* < 0.05).

**Table 1:** Basic characteristics of participants.

	Hippotherapy	WBV	p value
	Mean ± SD	Mean ± SD	
**Age (years)**	4.25 ± 0.7	4.26 ± 0.61	0.92
**Sex**			
Male	17 (56.7%)	15 (50%)	0.6
Female	13 (43.3%)	15 (50%)	

SD, standard deviation.

### Efficacy of both applications in ameliorating abdominal muscle thickness and GMFM-88

A significant interaction between treatment and time was identified with mixed MANOVA (F = 42.28, *p* = 0.001). The main effect of time was significant (F = 360.3, *p* = 0.001). The main effect of treatment was significant (F = 17.78, *p* = 0.001).

### Within group comparison

A significant increase in abdominal muscle thickness was observed for the EO, IO, TA, and RA in the hippotherapy and WBV groups after treatment (*p* > 0.001). In addition, there was a significant increase in GMFM-88 after treatment in the hippotherapy and WBV groups (*p* > 0.001; Table 2).

**Table 2:** Mean abdominal muscle thickness and GMFM-88 before and after treatment in the hippotherapy and WBV groups.

	Hippotherapy	WBV	MD (95% CI)	p value
	Mean ± SD	Mean ± SD		
**Abdominal muscle thickness (cm)**				
**EO**				
Pretreatment	0.300 ± 0.023	0.299 ± 0.024	0.003 (−0.009:0.01)	0.62
Post-treatment	0.390 ± 0.028	0.354 ± 0.031	0.036 (0.02: 0.51)	**0.001**
MD (95% CI)	−0.09 (−0.096: −0.08)	−0.055 (−0.063: −0.047)		
	***p = 0.001***	***p = 0.001***		
**IO**				
Pretreatment	0.417 ± 0.029	0.411 ± 0.019	0.006 (−0.005:0.016)	0.27
Post-treatment	0.520 ± 0.031	0.456 ± 0.026	0.064 (0.049: 0.079)	**0.001**
MD (95% CI)	−0.103 (−0.112: −0.094)	−0.045 (−0.054: −0.036)		
	***p = 0.001***	***p = 0.001***		
**TA**				
Pretreatment	0.322 ± 0.021	0.314 ± 0.022	0.008 (−0.003:0.019)	0.16
Posttreatment	0.434 ± 0.026	0.383 ± 0.025	0.051 (0.038: 0.064)	**0.001**
MD (95% CI)	−0.112 (−0.119: −0.105)	−0.069 (−0.076: −0.061)		
	***p = 0.001***	***p = 0.001***		
**RA**				
Pretreatment	0.521 ± 0.023	0.511 ± 0.025	0.01 (−0.003:0.023)	0.12
Posttreatment	0.691 ± 0.049	0.572 ± 0.036	0.119 (0.096: 0.141)	**0.001**
MD (95% CI)	−0.17 (−0.181: −0.159)	−0.061 (−0.073: −0.05)		
	***p = 0.001***	***p = 0.001***		
**GMFM-88**				
Pretreatment	45.73 ± 3.96	47.02 ± 3.08	−1.29 (−3.12:0.54)	0.16
Posttreatment	55.56 ± 4.18	52.7 ± 3.58	2.86 (0.85: 4.87)	**0.006**
MD (95% CI)	−9.83 (−10.54: −9.11)	−5.68 (−6.39: −4.96)		
	***p = 0.001***	***p = 0.001***		

CI, confidence interval; EO, external oblique; IO, internal oblique; MD, mean difference; RA, rectus abdominis; SD, standard deviation; TA, transversus abdominis; WBV, whole-body vibration.

### Between group comparison

No significant differences in all parameters were found between both groups pre-treatment (*p* > 0.05). Comparison between groups after treatment revealed a significantly greater increase in the abdominal muscle thickness of the EO, IO, TA, and RA in the hippotherapy group compared with the WBV group (*p* > 0.001). In addition, a significantly greater improvement was observed in GMFM-88 in the hippotherapy group than the WBV group (*p* > 0.01; Table 2).

## Discussion

The current study aimed to compare the effects of hippotherapy and WBV in improving sitting and abdominal muscle thickness in children with diplegia. Our results indicated significant improvements in sitting function and abdominal muscle thickness in both groups, but the hippotherapy group (B) showed greater progress than the WBV group (A). Achieving sitting posture control is critical for children with CP to improve their quality of life.^33^^,^^34^

The enhancement in both groups might have been a result of the effects of the conventional physical therapy program in ameliorating delayed milestones, particularly equilibrium, sitting, and standing, through stimulating the abdominal muscles and improving sitting posture. The significant improvement in both groups was consistent with findings reported by Bobath^35^ and Veerle et al.,^36^ who have proposed that the principal intention to enhance motor abilities and strength may be achieved through a regular physical therapy program that strengthens the abdominal and trunk muscles. In addition, our results are consistent with those of Ali et al.,^31^ who have suggested that a trunk and lower extremity muscle strengthening program followed by WBV enhances motor function in children with diplegia.

The results of Bogaerts et al. also supported our findings; in that study, WBV training has been found to improve muscle strength and affect the development of power, thus improving gross motor function.^37^ We concluded that WBV improves the strength of abdominal muscles and subsequently increases muscle mass. In addition, Cawthon et al. have established a significant association between lean body mass and muscle strength. Consequently, WBV may have beneficial effects on muscle mass.^38^ The findings of the present research, in agreement with prior studies, support that abdominal muscle strength can be achieved in children with spastic diplegia.^39^

Our results regarding the effect of WBV on motor function are reinforced by a prior study^40^ demonstrating that WBV effectively enhances motor function, balance, and gait in children with CP. In addition another study^41^ has reported that WBV for 12 weeks improves motor performance in standing and walking, and increases walking speed and muscle strength.

Because children with CP frequently lack control of their trunk muscles, the primary goal of therapy is to achieve anticipatory and reactive postural stability.^42^ Thus, hippotherapy is a strategy to improve postural stability and also develop enactive adjustment of posture, which is crucial in CP.

Current studies have confirmed that hippotherapy is an effective method and a therapy modality that increases gross motor function in children with spastic CP.^43^, ^44^, ^45^, ^46^ In addition, McGibbon et al. have established that gross motor function, and thus energy expenditure during walking in CP, may improve with hippotherapy.^45^

According to Cherng et al., hippotherapy can help children with spastic CP improve their gross motor function and should be continued for at least 16 weeks.^46^

GMFM-88 and GMFCS may provide data for service planning and therapy outcome documentation in children with CP.^47^^,^^48^ Therefore, many studies of hippotherapy have used GMFM-88 and GMFCS to assess which intervention methods work best for children with CP and to measure the therapy outcomes.^45^^,^^49^^,^^50^

Hippotherapy may be a realistic option to improve functional outcomes in children with CP.^50^ Casady and Nichols-Larsen,^51^ in a study of ten children with CP 2.3–6.8 years of age have observed improved scores of GMFM-88 after 10 weeks of a rehabilitation program. In contrast, Davis et al.^52^ have reported that GMFM-88 scores were not substantially changed after hippotherapy.

Sterba et al. (2002) have hypothesized that the rider's pelvic movement improves as a result of the horse's movement, thus resulting in a more functional gait. Additionally, forward and backward rocking motions stimulate the trunk muscles through an autonomic reaction that enhances pelvic tilting (anterior/posterior) and improves trunk stability.^43^

The major goals of hippotherapy are to enhance postural control and stimulate normal development and motor functional recovery in children with neurological diseases, primarily CP.^53^^,^^54^ Hippotherapy also causes the trunk muscles to contract faster to keep the rider balanced when the horse moves in three dimensions.

Hodges and Richardson^55^ have stated that the movement of the trunk muscles is essential for pelvic and lower extremity movement and maintaining postural stability; notably, the TA is the most rapidly contracting abdominal muscle. Hide et al.^56^ have suggested that the IO, EO, and TA increase trunk stabilization and postural control, and the TA is the most essential of these muscles. Therefore, the three-dimensional trunk swaying during horseback riding continually activates the transverse muscles.

The results of hippotherapy treatment on abdominal thickness are consistent with those in a prior study^57^ reporting substantially higher TA levels after hippotherapy.

## Limitations of the study

The effects of psychological factors associated with the treatment programs were not evaluated.

## Conclusion

The current study revealed a significant increase in abdominal thickness in both groups, and enhanced sitting function as a result of greater trunk muscle strength, after a regular physical therapy program. Hippotherapy and WBV can effectively enhance abdominal muscle thickness and improve sitting performance in children with diplegic CP, but hippotherapy is better than WBV according to our results. Therefore, we recommend hippotherapy as a better option for improving abdominal muscle thickness and achieving daily living tasks in children with diplegic CP.

## Recommendations

This study shows that hippotherapy and WBV can help increase abdominal muscle thickness and improve sitting performance in children with diplegic CP. According to these findings, we recommend that this treatment be applied to other types of CP, to compare the results and determine which types experience the best effects. The findings of this study may be useful for children with CP to improve problems with trunk control or sitting function.

## Source of funding

This study did not receive any specific grants from funding agencies in the public, commercial, or not-for-profit sectors.

## Conflict of interest

The authors have no conflict of interest to declare.

## Ethical approval

The Ethical Committee of the Faculty of Physical Therapy at Cairo University approved this study (No: P.T.REC/012/003211) on December 1, 2020.

## Authors contributions

MSM conceived and designed the study, conducted research, provided research materials, and collected and organized the data. ASA analyzed and interpreted data, wrote the initial and final drafts of the article, and provided logistic support. All authors have critically reviewed and approved the final draft and are responsible for the content and similarity index of the manuscript.
